# Activated hippo signal pathway inhibits cell proliferation and promotes apoptosis in NK/T cell lymphoma cells

**DOI:** 10.1002/cam4.2174

**Published:** 2019-05-23

**Authors:** Yu Chang, Xiao‐Rui Fu, Meng Cui, Wei‐Ming Li, Lei Zhang, Xin Li, Ling Li, Zhen‐Chang Sun, Xu‐Dong Zhang, Zhao‐Ming Li, Xiao‐Yan You, Fei‐Fei Nan, Jing‐Jing Wu, Xin‐Hua Wang, Ming‐Zhi Zhang

**Affiliations:** ^1^ Department of Oncology The First Affiliated Hospital of Zhengzhou University Zhengzhou China; ^2^ Department of Head & Neck and Thyroid The Cancer Hospital Affiliated to Zhengzhou University Zhengzhou China; ^3^ Department of Oncology Henan University of Chinese Medicine Zhengzhou China; ^4^ Department of Obstetrics and Gynecology Zhengzhou Central Hospital Affiliated to Zhengzhou University Zhengzhou China

**Keywords:** apoptosis, cell proliferation, hippo signaling pathway, NK/T cell lymphoma, YAP

## Abstract

**Background:**

Natural Killer T–Cell Lymphoma (NKTCL) is a subtype of Non‐Hodgkin's Lymphoma, and its morbidity is ranked the first of T‐Cell Lymphoma. Hippo signaling pathway is involved in the pathogenesis of tumors. However, the role of Hippo signaling pathway in the oncogenesis of NKTCL still remains unclear.

**Methods:**

The expressions of mammalian sterile 20‐like kinase 1 (MST1) and Yes‐associated protein (YAP) were investigated by RT‐PCR and Western blotting. Cell viability was detected by MTT assays. Cell cycle and cell apoptosis were determined by flow cytometry. Cell proliferative capacity was detected by colony formation assay. Nude mice xenograft models were established and the tumor sections were analyzed by immunohistochemistry (IHC) staining.

**Results:**

The expression of MST1 was significantly down‐regulated in NKTCL tissues (n = 30) and cell lines, while the expression of YAP was significantly up‐regulated, and the phosphorylation of YAP was inhibited. Overexpression of MST1, knockdown of YAP, or verteporfin (VP) treatment could inhibit cell proliferation, and promote cell cycle arrest and apoptosis in NKTCL cells, while knockdown of MST1 and overexpression of YAP promoted cell proliferation. Additionally, Bcl‐2/Bax ratio and downstream effectors of Hippo signaling pathway (c‐myc, survivin, cyclinD1, CTGF, and TEAD) were significantly decreased when MST1 was overexpressed and YAP was knocked down or after VP treatment. Furthermore, our mice model demonstrated that activation of Hippo signal pathway suppressed the tumorigenesis of NKTCL.

**Conclusion:**

The activation of Hippo signal pathway via overexpressing MST1 or down‐regulating YAP can inhibit the tumorigenesis of NKTCL.

## 1 INTRODUCTION

Malignant lymphoma (ML) is a type of malignant tumor that stems from lymph gland and/or extranodal lymphoid tissue, and its morbidity is showing a trend of continuous growth worldwide, becoming a disease that severely impairs human health.^1^ ML can be classified into Hodgkin's Disease (HD) and Non‐Hodgkin's Lymphoma (NHL) according to the histology and pathological pattern. Natural Killer T–Cell Lymphoma (NKTCL) is a subtype of NHL of which the morbidity is ranked the first of T‐Cell Lymphoma.^2^ NKTCL has a unique epidemiological distribution and commonly occurred in East Asia and Latin America.^3^ CHOP (cyclophosphamide, hydroxydaunorubicin, oncovin, prednisone) is a frequently used chemotherapy regimen for NKTCL, but the effect is far from satisfactory.^4^ Nowadays, it is more common to employ regimens that incorporate radiotherapy and chemotherapy. Lin et al^5^ and Michot et al^6^ increased the 2‐year survival rate of extranodal NKTCL patients to 80.1% and 72% by employing radiotherapy and chemotherapy. However, given the high malignancy grade, rapid clinical progression and poor long‐term prognosis of extranodal NKTCL, exploring the underlying mechanism of NKTCL is of great significance for the development of drugs for NKTCL.

Signaling pathways that regulate the signals of cell growth and development usually play an important role in tumorigenesis and tumor progression. Currently, there are studies showing that the oncogenesis of extranodal NKTCL is closely related to signaling pathways such as NF‐κB, JAK‐STAT, platelet derived growth factor (PDGF), and NOTCH‐1, among which, NF‐κB and JAK/STAT are the best studied in NKTCL. NF‐κB signaling pathway is extensively involved in the regulation of cell proliferation and development of multiple immune cells.^7^ Studies showed that NF‐κB was up‐regulated in the primary tumor cells of extranodal NKTCL caused by DDX3X (ATP‐dependent RNA helicase) mutation.^8^ These data proved that the continuous activation of the NF‐κB signaling pathway indicated potential carcinogenesis. JAK‐STAT signaling pathway also plays an important role in the pathogenic mechanism of extranodal NKTCL and it shows potential value of targeted therapy. In 2014, Bouchekioua et al^9^ conducted Sanger sequencing on 24 exons of JAK3, and confirmed three cases of 19 patients suffered JAK3 A573V mutation and one suffered JAK3 V722L mutation. However, some studies on cases of extranodal NKTCL failed to detect any JAK3 genetic mutation.^10^, ^11^ The mutation rates of JAK3 in different studies are greatly varied with a relatively low level, so studies of large samples on its pathogenic mechanism are yet to be conducted for verification.

Recently, the Hippo signaling pathway has been reported to promote cell death and differentiation and inhibit cell proliferation.^13^, ^14^ More and more evidences show that Hippo signaling pathway is involved in the oncogenesis of a variety of tumors, such as carcinomas of the lung, pancreas, esophagus, liver, and mammary gland (reviewed in Pan^15^). Hippo signaling pathway is originally found inside drosophila, which can exert significant regulatory effect on the size of organs.^13^ This pathway involves mammalian sterile 20‐like kinases 1/2 (MST1/2), Sav1, large tumor suppressor 1/2 (LATS1/2), MOB1 (MOBKL1A/MOBKL1B), yes‐associated protein (YAP)/transcriptional coactivator with PDZ‐binding motif (TAZ) and TEAD1‐4.^13^, ^16^ MST1/2, Sav1, LATS1/2, and MOB1 constitute the core kinase chain of the Hippo signaling pathway of mammals.^13^, ^16^ When Hippo signaling is inactivated, YAP is in an activated state, entering cell nucleus and integrating with transcription factor TEAD, thus inducing a series of genetic expressions related to cell proliferation; when Hippo signaling pathway is activated, MST1/2 phosphorylates MOB1 and LATS1/2 and strengthens their interaction with the assistance of scaffolding protein Sav1, so as to activate the phosphorylated LATS1/2.^13^, ^16^ Then, the activated LATS1/2 further phosphorylates the transcriptional coactivator YAP/TAZ which can be sequestered in the cytoplasm and degraded by ubiquitination, hence obstructing the expression of downstream targeted genes.^13^, ^14^, ^16^

As reported, MST1 and YAP in the Hippo signaling pathway are differentially expressed in a variety of tumors, thereby participating in the regulation of multiple tumorigeneses. Previous study illuminated that MST1 was highly expressed in hematopoietic cells, and knockdown of MST1 could cause lymphoma development by inducing chromosomal instability.^17^ YAP played a carcinogenic role in a variety of tumors, including prostate cancer and head and neck squamous cell carcinoma.^18^, ^19^ Overexpression of YAP could facilitate cell proliferation and migration of prostatic epithelial cells, as well as the vicious transformation of epithelial cells.^18^ Furthermore, YAP acted as a tumorigenic factor in bladder cancer cells, and the highly expressive YAP promoted cell growth and migration of such tumor cells,^20^, ^21^ suggesting that Hippo signaling pathway is seen as a new potential channel that is involved in the pathogenesis of various tumors. However, the role of Hippo signaling pathway in the oncogenesis of NKTCL still remains unclear.

In this study, we found that the activation of Hippo signal pathway via overexpressing MST1 or down‐regulating YAP can inhibit the tumorigenesis of NKTCL. This study is conducive for us to better understand the pathogenesis of NKTCL, and discover key molecular markers during the progression of NKTCL, providing novel insights for targeted therapy of NKTCL.

## 2 MATERIALS AND METHODS

### 2.1 Clinical specimens and cell culture

Thirty pairs of NK/T Cell Lymphoma samples and corresponding normal tissues were collected from the oncology department of the First Affiliated Hospital of Zhengzhou University. NKTCL was diagnosed according to the 2008 World Health Organization classification.^22^ Written informed consent was obtained from all patients. All tissue samples were stored at −80°C until use. The human NKTCL cell lines SNK‐6 and YTS, and normal NK cells (ATCC) were cultured in RPMI‐1640 (Gibco) supplemented with 10% fetal bovine serum (FBS; Gibco) and 700 U/mL interleukin‐2 (IL‐2), and incubated at 37°C, and 5% CO_2_. All the experiments were approved by the Ethics Committees of the First Affiliated Hospital of Zhengzhou University.

### 2.2 RNA extraction and RT‐PCR

RNA was isolated from tissue samples and cell lines with TRIzol reagent (Invitrogen) and RNeasy Plus Micro Kit (QIAGEN) according to the manufacturer's instructions. Then, reverse transcription was conducted to synthesize the cDNA by utilizing the SuperScript® IV First‐Strand Synthesis System (Invitrogen). RT‐PCR was performed in Applied Biosystems 7500 Real Time PCR System (Applied Biosystems), using 20 ng template in 25 μL reaction volume with 2× Power SYBR® Green PCR Master Mix (Invitrogen) and gene specific primer pairs for MST1 (F: 5′‐AGACCTCCAGGAGATAATCAAAGA‐3′; R: 5′‐AGATACAGAACCAGCCCCACA‐3′), YAP (F: 5′‐ACCCACAGCTCAGCATCTTCG‐3′; R: 5′‐TGGCTTGTTCCCATCCATCAG‐3′), and β‐actin (F: 5′‐ CCTCGCCTTTGCCGATCC‐3′; R: 5′‐ GGATCTTCATGAGGTAGTCAGTC‐3′) in NKTCL cell lines and NKTCL tissue samples homogenates. Amplification conditions were as follows: 95°C for 10 minutes followed by 45 cycles consisting of 95°C for 15 seconds, 60°C for 30 seconds and 68°C for 60 seconds. The gene expression levels for all samples were normalized to β‐actin mRNA expression using the comparative Ct method. Relative gene expression was calculated using 2^−ΔΔCt^ method. All data are displayed as the mean ± SD of three independent experiments.

### 2.3 Cell transfection

Yes‐associated protein and MST1 specific short hairpin RNAs (shRNA) were cloned into GV118 vector. The targeted sequences are as follows: shYAP, 3′‐GACATCTTCTGGTCAGAGA‐5′; shMST1, 5′‐GGGCACTGTCCGAGTAGCAGC‐3′; scrambled control RNA interference sequence, 3′‐GACATTTGTAACGGGATTC‐5′. NKTCL cells were transfected with lentiviral shYAP or shMST1 using Lipofectamine LTX reagent (Thermo Fisher Scientific) to package the shYAP or shMST1‐containing lentivirus. The lentivirus production and infection were performed as described previously.^23^ For MST1 and YAP overexpression, NKTCL cells were transfected with lentivirus containing a plasmid encoding MST1 and YAP using Lipofectamine 2000 (Thermo Fisher Scientific).

### 2.4 Verteporfin (VP) treatments

Verteporfin (Selleckchem) was dissolved in dimethyl sulfoxide (DMSO), and diluted to desired concentrations in phosphate‐buffered saline (PBS) before use. For in vitro study, NKTCL cells were treated with 0, 1, 2, 4, 6, 8 μmol/L verteporfin or DMSO for 24, 48, or 72 hours at 37°C, 5% CO_2_, respectively, under the condition of darkness during both treatment and lysis. For in vivo study, mice were administered intraperitoneally (IP) at a dose of 100 mg/kg every 2 days for total 3 weeks, and the control mice were administered with equivalent PBS.

### 2.5 MTT assay

Cell viability of NKTCL cells was detected using MTT assay (Sigma). Cells were cultured onto 96‐well plates with 100 μL of growth medium in a humidified atmosphere (37°C, 5% CO_2_). After culturing for 24 hours, the medium was replaced with fresh culture medium containing 10 μL of MTT dye (final concentration 0.5 mg/mL). After 4 hours incubation at 37°C, the MTT solution was replaced with 150 μL of DMSO. The absorbance at 490 nm was then detected with a microplate reader (BioTek).

### 2.6 Flow cytometry analysis of cell cycle and apoptosis

Apoptotic cells were identified using the Propidium Iodide (PI) Flow Cytometry Kit (abcam) and FITC Annexin V Apoptosis Detection Kit I (BD) according to the manufacturer's instructions. NKTCL cells were transfected for 24 hours and then cultured for 72 hours in a humidified atmosphere (37°C, 5% CO_2_) before determining the cell cycle and the extent of apoptosis. The cells were collected, washed twice with cold PBS and resuspended in 1× binding buffer. For cell cycle analysis, the cells were stained with 5 μL PI for 10 minutes in the dark at room temperature. For the cell apoptosis analysis, the cells were stained with 5 μL annexin v‐FITC for 15 minutes and then 5 μL PI for 10 minutes in the dark at room temperature. The cells were examined using a FACSCanto II flow cytometer (BD Biosciences).

### 2.7 Soft agar colony formation assay

Soft agar colony formation assay was applied to detect the proliferative capacity of NKTCL cells after transfections and VP treatment. The assay was performed as previously described.^24^ six‐well culture plates containing the bottom and soft layers were used. NKTCL cells were plated in six‐well plates. After incubation for 2 weeks in culture medium with 10% FBS, the medium was removed. Cells were fixed and stained with crystal violet dye for 1 hour. The images of colonies were photographed, and the number of colonies was counted using ImageJ.

### 2.8 Nude mice xenograft experiments

The animal experiments were approved by the Ethics Committees of the First Affiliated Hospital of Zhengzhou University. Six‐week‐old male BALB/c nude (nu/nu) mice (n = 40) were purchased from SJA Laboratory Animal Co., Ltd (Hunan). The mice were randomly divided into five groups: (a) SNK6 cells and 100 mg/kg VP by intraperitoneal (IP) injection (VP group); (b) SNK6 cells transfected with sh‐YAP (sh‐YAP group); (c) SNK6 cells transfected with plasmids encoding MST1 (MST1 group); (d) SNK6 cells transfected with empty plasmids (vector group); (e) PBS (control group). Four hundred microliters of cells suspension (about 1 × 10^7^) in PBS were intraperitoneally injected into the nude mice. For the VP treatment, when tumors reached a mean volume of 70‐150 mm^3^, the mice in the VP group were intraperitoneally injected with VP (100 mg/kg body weight) every 2 days for 3 weeks. Tumor sizes were measured every 3 days with electronic caliper. Tumor volume (*V*) was calculated by the formula: *V* = 0.5 × length × width^2^. All mice were sacrificed at day 30 after treatments. Tumor samples were collected and weighed for all groups.

### 2.9 Immunohistochemistry (IHC) staining

Xenograft tumor samples were collected and fixed in 4% paraformaldehyde, then transferred to 70% ethanol and embedded in paraffin. Sections were cut from paraffin blocks into 4 μm thick sections and mounted on charged slides. The tissue sections were deparaffinized with dimethylbenzene and rehydrated after being baked at 60°C for 2 hours. Antigen retrieval was subsequently performed, and 3% hydrogen peroxide was used to quench peroxidase activity for 20 minutes. The slides were incubated with mouse monoclonal anti‐YAP (1:100; Cell Signaling Technology) overnight at 4°C. The complex was visualized with DAB complex, and the nuclei were counterstained with haematoxylin. The staining intensity of YAP was quantified.

### 2.10 Western blot analysis

Cells were harvested and lysed in the RIPA buffer (Sigma‐Aldrich). Protein concentrations were determined using the BCA protein assay kit (Thermo Fisher Scientific). Proteins (30 μg) were separated by 10% SDS‐PAGE and transferred onto a nitrocellulose membrane. After blocking with 5% BSA, the membranes were then incubated with primary antibodies against MST1, YAP, p‐YAP, c‐myc, survivin, cyclinD1, CTGF, Bcl‐2, Bax, and GAPDH (1:1000; Abcam). GAPDH was loaded as an internal reference. Bands were then treated with horseradish peroxidase‐conjugated secondary antibodies (Abcam) at room temperature for 2 hours. The signal was developed using the ECL system (Beyotime) according to the manufacturer's instructions. The relative protein expression was analyzed by NIH Image J software and presented as the density ratio to that of the internal reference.

### 2.11 Statistical analysis

Data were analyzed with Prism 5.0 (GraphPad Software). All data were expressed as the means ± standard deviation (SD). One‐way analysis of variance (ANOVA) with multiple comparisons using Dunnett's test was applied to compare the difference between groups. *P* < 0.05 was considered significantly different.

## 3 RESULTS

### 3.1 Expression levels of MST1 and YAP in NKTCL tissues and cell lines

**Figure 1:**
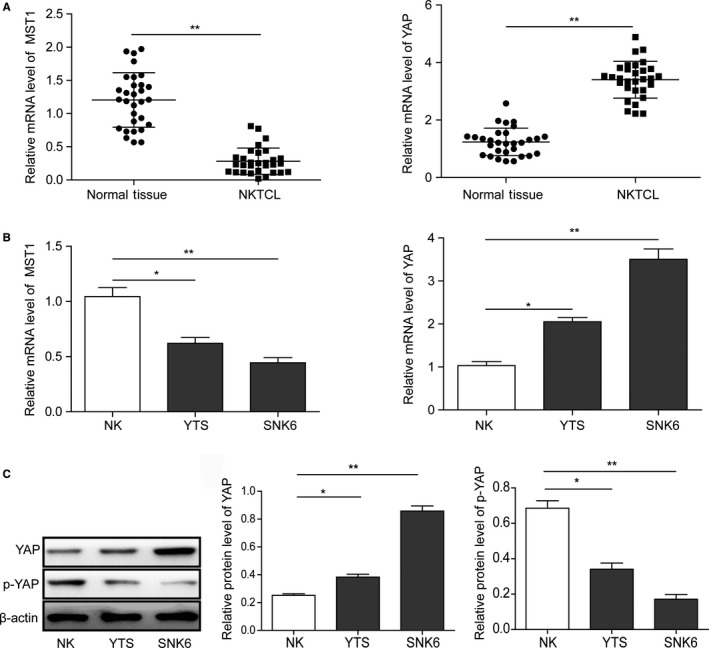
Expression levels of MST1 and YAP in NKTCL tissues and cell lines. (A) The expressions of MST1 and YAP in 30 pairs of NKTCL samples and corresponding normal tissues, and (B) NKTCL cell lines (SNK6 and YTS) detected by RT‐PCR. (C) Western blot analysis of the protein expression levels of YAP and phosphorylated YAP in NKTCL cell lines. All the results were shown as mean ± SD (n = 3), which were three separate experiments performed in triplicate. *P < 0.05. **P < 0.01

The expression levels of MST1 and YAP in 30 pairs of NKTCL samples and corresponding normal tissues, and NKTCL cell lines (SNK‐6 and YTS) were investigated by RT‐PCR. As shown in Figure 1A, the expression of MST1 in NKTCL tissues was significantly lower than that in the normal tissues (*P* < 0.01), while the expression of YAP in NKTCL tissues was significantly higher than that in the normal tissues (*P* < 0.01). Consistently, the mRNA expression levels of MST1 and YAP were down‐regulated (*P* < 0.01) and up‐regulated (*P* < 0.01), respectively, in NKTCL cell lines compared with the normal NK cells (Figure 1B). Moreover, the Western blot analysis showed that the protein expression level of YAP was up‐regulated in NKTCL cell lines (*P* < 0.01), while the phosphorylated YAP was significantly down‐regulated (Figure 1C; *P* < 0.05, vs the normal NK cells). All these results indicate that the Hippo signal pathway is inhibited in NKTCL tissues and cell lines, thus blocking the phosphorylation of YAP.

### 3.2 Effects of MST1 and YAP on the proliferation of NKTCL cells

**Figure 2:**
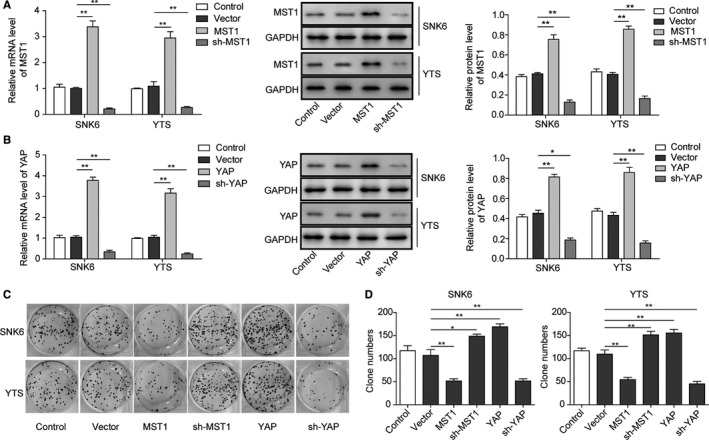
Effects of MST1 and YAP on the proliferation of NKTCL cells. The mRNA and protein levels of MST1 (A) and YAP (B) were overexpressed or down‐regulated in both SNK6 and YTS cell lines after transfection of MST1 or YAP encoding plasmid and sh‐MST1 or sh‐YAP determined by RT‐PCR and Western blotting. (C) Cell proliferation determined by soft agar colony formation assay after transfection of MST1 or YAP encoding plasmids and sh‐MST1 or sh‐YAP. (D) Quantification of the colony number in (C). All the results were shown as mean ± SD (n = 3), which were three separate experiments performed in triplicate. *P < 0.05. **P < 0.01

In order to further investigate the role of MST1 and YAP in NKTCL tumorigenesis, sh‐YAP or sh‐MST1 and plasmids encoding YAP or MST1 were used to knockdown or overexpress YAP and MST1, respectively, in NKTCL cells (SNK6 and YTS cells). As shown in Figure 2A,B, MST1 and YAP were up‐regulated or down‐regulated at both mRNA and protein levels in SNK6 and YTS cell lines after the transfections (Figure 2A,B; *P* < 0.05, vs the vector group). Additionally, the numbers of cell colonies formed by MST1 plasmid or sh‐YAP transfected NKTCL cells were significantly less than those by the vector transfected cells (*P* < 0.01; Figure 2C,D). Compared with the vector group, the colony formation was enhanced by MST1 knockdown and YAP overexpression (Figure 2C,D). All these results indicate that overexpression of MST1 or knockdown of YAP can inhibit the NKTCL cell proliferation.

### 3.3 Effects of MST1 and YAP on the cell cycle and apoptosis of NKTCL cells

**Figure 3:**
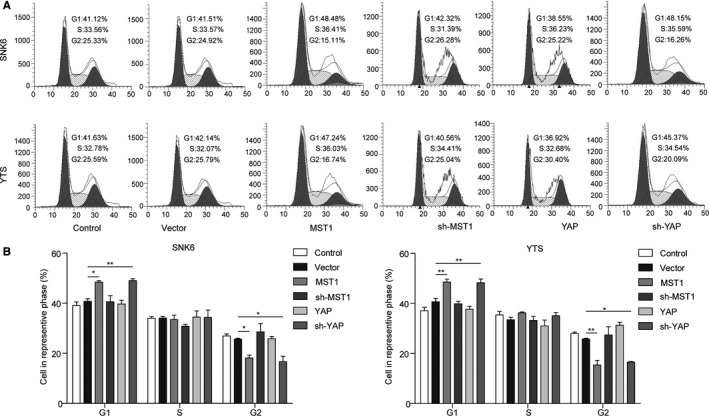
Effects of MST1 and YAP on cell cycle of NKTCL cells. (A) Cell cycle was detected by flow cytometry analysis in both SNK6 and YTS cell lines transfected with MST1 or YAP encoding plasmid and sh‐MST1 or sh‐YAP determined. (B) Cell number in G1, S, and G2 stages for different groups. All the results were shown as mean ± SD (n = 3), which were three separate experiments performed in triplicate. *P < 0.05. **P < 0.01

**Figure 4:**
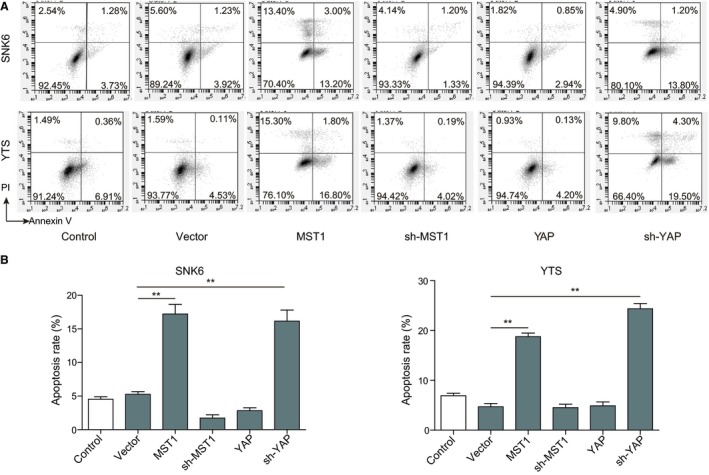
Effects of MST1 and YAP on cell apoptosis of NKTCL cells. (A) Cell apoptosis was detected by flow cytometry analysis in both SNK6 and YTS cell lines transfected with MST1 or YAP encoding plasmid and sh‐MST1 or sh‐YAP determined. (B) Quantification of apoptosis rate in (A). All the results were shown as mean ± SD (n = 3), which were three separate experiments performed in triplicate. *P < 0.05. **P < 0.01

The effects of overexpression or knockdown of MST1 and YAP on cell cycle and apoptosis of NKTCL cells were further investigated. Consistently, cell cycle analysis showed that the ratio of NKTCL cells at G1 was significantly increased when the MST1 was overexpressed (*P* < 0.05, vs the vector group) or YAP was knocked down (*P* < 0.01, vs the vector group), while YAP overexpression and MST1 knockdown exhibit no significant effects on the cell cycle progression of NKTCL cells compared with the vector group (Figure 3A,B). Moreover, the flow cytometry analysis (Figure 3B) revealed a significantly higher percentage of apoptotic cells in both NKTCL cell lines when the MST1 was overexpressed or YAP was knocked down (*P* < 0.01, vs the vector group; Figure 4A,B). Consistently, both YAP overexpression and MST1 knockdown exhibit no significant effects on the apoptosis of NKTCL cells when compared with the control group (Figure 4A,B). All these results indicate that overexpression of MST1 or knockdown of YAP can inhibit the NKTCL cell cycle progression and enhance the cell apoptosis.

### 3.4 VP treatment inhibits proliferation and enhances apoptosis of NKTCL cells via inhibiting the YAP expression

**Figure 5:**
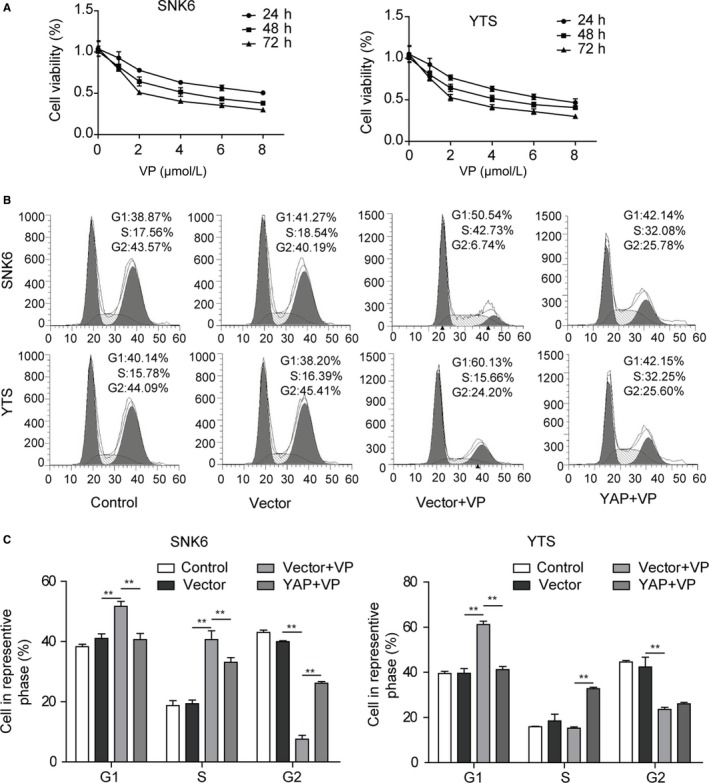
Verteporfin (VP) treatment inhibits cell viability and cell cycle by repressing YAP in NKTCL cells. (A) Cell viability was assessed by MTT assay in NKTCL cells treated with VP or transfected with YAP encoding plasmid. (B) Cell cycle was measured by flow cytometry analysis in NKTCL cells treated with VP or transfected with YAP encoding plasmid. (C) Cell number in G1, S and G2 stages for different groups. All the results were shown as mean ± SD (n = 3), which were three separate experiments performed in triplicate. *P < 0.05. **P < 0.01

**Figure 6:**
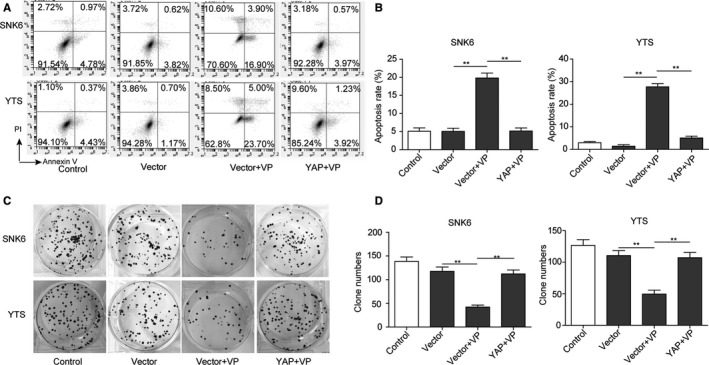
Verteporfin (VP) treatment promotes apoptosis and inhibits cell proliferation by repressing YAP in NKTCL cells. (A) Cell apoptosis was measured by flow cytometry analysis in NKTCL cells treated with VP or transfected with YAP encoding plasmid. (B) Quantification of apoptosis rate in (A). (C) Cell proliferation was detected by soft agar colony formation assay in NKTCL cells treated with VP or transfected with YAP encoding plasmid. (D) Quantification of the colony number in (C). All the results were shown as mean ± SD (n = 3), which were three separate experiments performed in triplicate. *P < 0.05. **P < 0.01

Verteporfin, a photosensitizer, was reported to inhibit YAP activation by disrupting YAP‐TEAD interactions and prevent YAP induced oncogenic growth.^25^ Therefore, VP was applied to treat the NKTCL cells in order to further investigate the effect of YAP on NKTCL tumorigenesis. As shown in the Figure 5A, treatment with VP reduced the viability of NKTCL cells in dose‐ and time‐dependent manners. Consistently, the ratio of NKTCL cells at G1 phase was significantly increased after VP treatment (Figure 5B,C; *P* < 0.01, vs the control group), indicating that the cell cycle progression was inhibited after the VP treatment. Moreover, the flow cytometry analysis revealed a significantly higher percentage of apoptotic cells in VP treated NKTCL cells (Figure 6A,B; *P* < 0.01, vs the control group). Additionally, the numbers of cell colonies formed by VP treated NKTCL cells were significantly less than those in the control group (*P* < 0.01; Figure 6C,D). To further confirm that the effects of VP on NKTCL cells were mediated by YAP, YAP was overexpressed in the VP‐treated NKTCL cells. As expected, overexpression of YAP was able to prevent apoptosis, cell cycle arrest, and proliferation inhibition mediated by VP treatment in NKTCL cells (*P* < 0.01, vs the vector + VP treated group; Figures 5 and 6). All these results demonstrate that VP inhibits cell proliferation while promotes cell apoptosis and cell cycle arrest by repressing YAP, suggesting that YAP plays a key role in the tumorigenesis of NKTCL.

### 3.5 Effects of Hippo signaling pathway on downstream effectors in NKTCL cells

**Figure 7:**
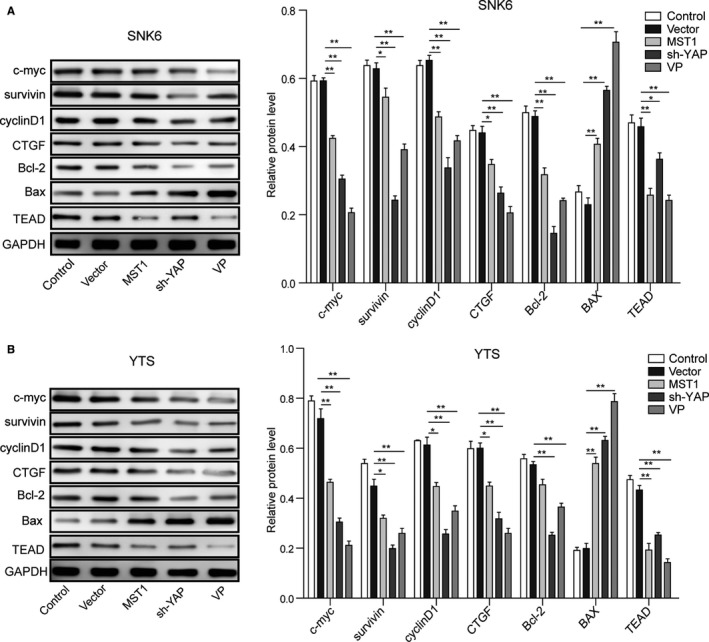
Expression levels of survival‐related proteins in NKTCL cell lines. (A) The protein levels of c‐myc, survivin, cyclinD1, CTGF, Bcl‐2, Bax, and TEAD in SNK6 cell were detected by Western blotting after transfection of MST1 plasmid and sh‐YAP and VP treatment. (B) The protein levels of c‐myc, survivin, cyclinD1, CTGF, Bcl‐2, Bax, and TEAD in YTS cell were detected by Western blotting after transfection of MST1 plasmid and sh‐YAP and VP treatment. All the results were shown as mean ± SD (n = 3), which were three separate experiments performed in triplicate. *P < 0.05. **P < 0.01

The downstream effector proteins of Hippo signaling pathway in NKTCL cells were detected by Western blotting. As shown in Figure 7, the expression levels of TEAD1, c‐myc, survivin, cyclinD1, CTGF, and Bcl‐2 in NKTCL cells were significantly down‐regulated (*P* < 0.05, vs the vector group), while Bax was significantly up‐regulated (*P* < 0.01, vs the vector group) when the MST1 was overexpressed and YAP was knocked down or after VP treatment. These results further confirm that MST1 overexpression or YAP down‐regulation can enhance apoptosis and inhibit cell proliferation in NKTCL.

### 3.6 Activation of Hippo signal pathway influences the tumorigenesis of NKTCL in mice

**Figure 8:**
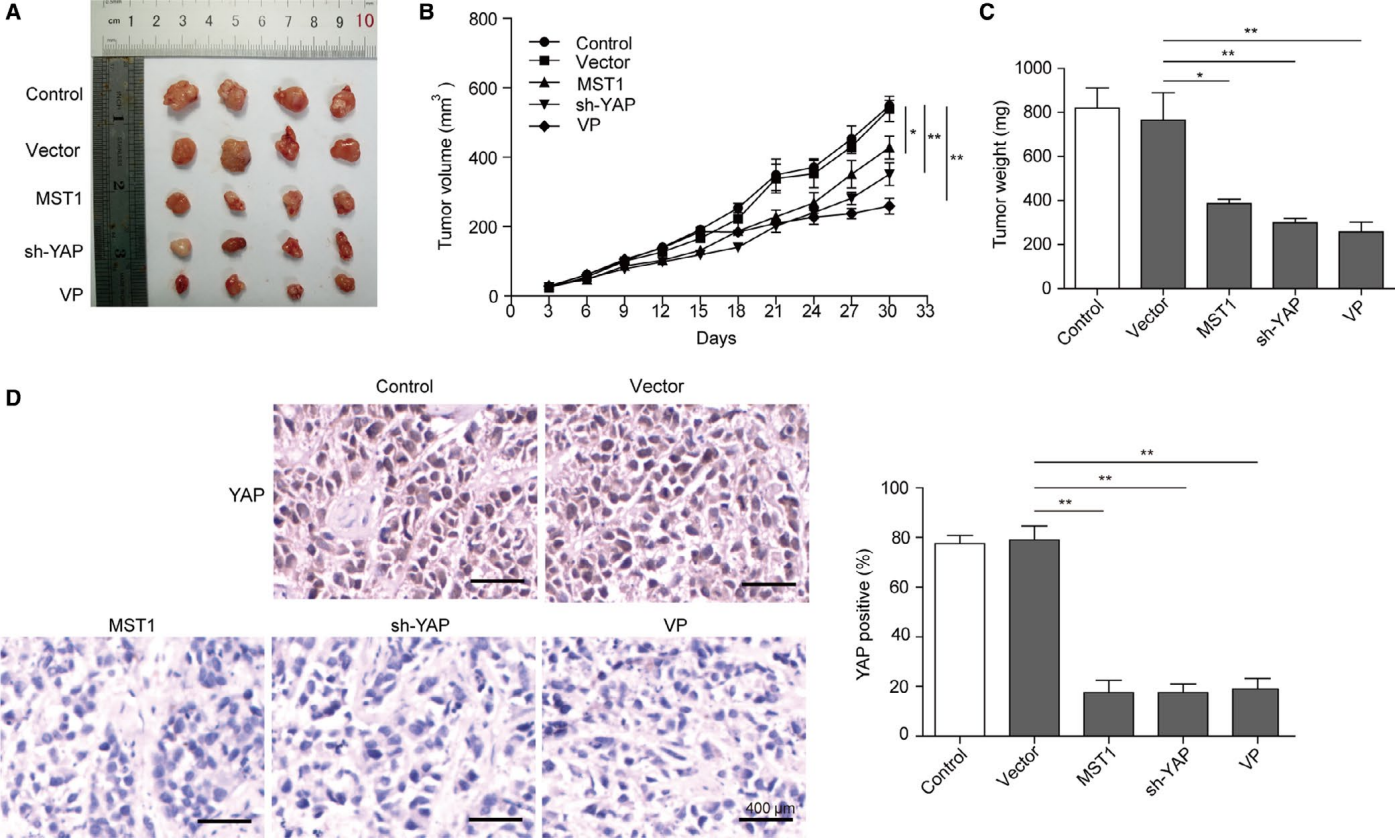
Activation of Hippo signal pathway influence the tumorigenesis of NKTCL in mice. (A) Tumors formed by nude mice xenograft experiments. (B) Tumor sizes measured every three days with electronic caliper. (C) Weights of tumors harvested from mice at the end of experiments. (D) Representative IHC images of YAP. Scale bar = 400 μm. All the results were shown as mean ± SD (n = 3), which were three separate experiments performed in triplicate. *P < 0.05. **P < 0.01

We next evaluated the influence of Hippo signal pathway on the tumorigenesis of NKTCL in mouse model. Tumors from MST1 overexpressed, YAP knocked down, or VP treated mice were significantly smaller than those from the vector or vehicle‐treated controls (Figure 8A,B). A same pattern was observed for the weights of tumors harvested from mice at the end of experiments (Figure 8C). Then, the IHC analysis (Figure 8D) of tumor sections showed that MST1 overexpression, YAP knockdown, or VP treatment significantly decreased the expression of YAP (*P* < 0.01) compared with the vector samples. These results further confirm that activation of Hippo signal pathway via overexpressing MST1 or down‐regulating YAP can inhibit the tumorigenesis of NKTCL.

## 4 DISCUSSION

Natural Killer T–Cell Lymphoma is a kind of aggressive Epstein‐Barr virus (EBV) infection‐related Non‐Hodgkin's lymphoma stemmed from mature NK cells and NK‐Like T Cells, and shows highly‐aggressive progression, poor prognosis and short patient survival time.^2^ Cascade amplification of abnormal signals in the upstream and downstream signaling pathways plays an extremely critical role in the oncogenesis and progression of tumors, so does the pathogeny of NKTCL. Many signaling pathways are found to be involved in the pathogeny of NKTCL, among which, NF‐κB and JAK/STAT are the best studied.^26^, ^27^ Studies showed that NF‐κB mutation was related to drug resistance among NKTCL patients.^28^ From 65 cases of NKTCL patients, Koo et al^29^ found 23 cases (35.4%) showed JAK3 mutation, indicating that JAK/STAT signaling pathway is involved in the oncogenesis and progression of NKTCL. Currently, the studies on mice models and human tumor tissues have shown that Hippo signaling pathway plays an important role in the oncogenesis of cancers.^15^ However, the underlying mechanisms of Hippo signaling pathway in NKTCL tumorigenesis is still unclear.

Hippo signaling pathway regulates proliferation and apoptosis of various cells by mediating multiple signaling kinase cascades.^13^ In a general way, this signaling pathway mainly consists of three correlated parts: upstream regulatory signal, Hippo core kinase cascade complex, and downstream intranuclear transcriptional regulatory complex.^16^ Hippo core kinase cascade complex includes a series of serine/threonine kinases, suppressing cell proliferation and facilitating cell apoptosis by phosphorylating YAP.^14^ The abnormality of Hippo upstream regulatory signals or core kinase cascade complex might lead to the organs overgrowth and the tumorigenesis.^13^, ^16^ Mice with liver‐specific deletions of Nf2, Sav1 and MST1/2 or overexpression of YAP ultimately suffered from liver cancer.^30^, ^31^ The abnormal regulations of Hippo signaling pathway were observed in many human tumors. For example, high expression or intranuclear enrichment of YAP/TAZ was detected in liver, mammary gland, lungs, and colon cancers.^32^, ^33^ In our study, the expression of MST1 was significantly down‐regulated in NKTCL tissues and cell lines, while the expression of YAP was significantly up‐regulated. Also, the phosphorylation of YAP was inhibited in both NKTCL tissues and cell lines. These indicate that the Hippo signal pathway is inhibited in NKTCL tissues and cell lines, thus blocking the phosphorylation of YAP.

MST1/2 is a kind of pro‐apoptosis kinase before Hippo signaling pathway is established, and it can be activated by cysteinyl aspartate‐specific proteinase.^34^ MST1/2 activates LATS1/2 through multiple mechanisms in the protein kinase cascade of Hippo signaling pathway.^35^, ^36^ MST1/2 can phosphorylate the C‐terminal hydrophobic group of LATS1/2, facilitating the self‐phosphorylation and activation of LATS1/2.^37^ MST1/2 also can phosphorylate MOB1 and facilitate the integration between the activity inhibitory areas of MOB1 and LATS1/2, thus removing the activity inhibition of LATS1/2 and making it activated.^37^, ^38^ The activated LATS1/2 further phosphorylates YAP which will be sequestered and degraded in the cytoplasm, hence obstructing the expression of downstream targeted genes. YAP plays an important role in the cell proliferation, survival, migration, and invasion, and the highly active YAP may help cells escape from cell contact inhibition and anoikis.^25^, ^39^ As reported, YAP was significantly up‐regulated in many tumors such as lung, oesophagus, bladder, and cervix (reviewed in Segrelles et al^19^). During the oncogenesis of pancreatic cancer, YAP mediated epithelial‐mesenchymal transition (EMT) and cancer cell proliferation.^40^ Additionally, MST1/2 deficiency resulted in cell overgrowth and hepatocellular carcinoma (HCC), and overexpression of MST1 and MST2 could suppress HCC development through inactivation of the YAP oncogene.^41^ The absence of Sav1 or MST1/2 may lead to bigger liver, causing the formation of tumors.^42^ In present study, we found that MST1 was down‐regulated while YAP was up‐regulated in NKTCL tissues and cells. Overexpression of MST1 and knockdown of YAP could inhibit cell proliferation and cell cycle progression, and promote cell apoptosis in NKTCL cells. These results fully indicate that activation of Hippo signaling pathway can inhibit the tumorigenesis of NKTCL, which was further confirmed in vivo in the nude mice xenograft model. The abnormality of Hippo signaling pathway is closely associated with multiple tumorigenesis.

Yes‐associated protein can be translocated to the nucleus and then regulate the activity of various transcription factors involved in cell proliferation, apoptosis, metastases development, and stem cell maintenance. In our study, overexpression of MST1 and knockdown of YAP could decrease the Bcl‐2/Bax ratio and other survival‐related proteins (c‐myc, survivin, cyclinD1, and CTGF) in NKTCL cells, thereby regulating the proliferation and apoptosis of NKTCL cells. YAP can interact with transcription factors, such as p73, SMADs, TBX5, AP‐1, and TEADs, or with transcriptional regulators such as the ICD of ERBB4 (reviewed in Segrelles et al^19^), among which TEADs are the main transcriptional factors mediating the tumorigenic effect of YAP.^13^, ^16^ Liu‐Chittenden et al^25^ discovered the porphyrin family, such as VP, hematoporphyrin (HP), and protoporphyrin IX (PPIX) can serve as YAP inhibitors to inhibit the interaction between YAP and TEADs. Studies proved that VP could eliminate liver overgrowth in vivo caused by YAP high‐expression or Nf2 inactivation.^25^ Brodowska et al^43^ found that VP inhibited the growth and survival ability of retinoblastoma through interfering YAP‐TEADs complex. Therefore, VP was recruited in this study to efficiently inhibit the YAP activation. The VP treatment inhibited the cell cycle progression and proliferation and increased apoptosis of NKTCL cells. Furthermore, overexpression MST1, knockdown of YAP, or VP treatment could decrease the expression level of TEAD, suggesting that Hippo signaling pathway affects proliferation, cycle and apoptosis of NKTCL cells by regulating YAP‐TEAD complex. All the above studies have shown that overexpression of MST1 or knockdown of YAP can activate the Hippo signal pathway, thus inhibiting the NKTCL cell proliferation and enhancing apoptosis.

In summary, our results demonstrated that Hippo signaling pathway was inhibited in NKTCL. The activation of Hippo signal pathway via overexpressing MST1 or down‐regulating YAP could inhibit cell proliferation while promote cell cycle arrest and apoptosis via regulating survival‐related signals by YAP‐TEAD complex in NKTCL cells, indicating that Hippo signaling pathway played a critical role in the tumorigenesis of NKTCL. MST1 and YAP are key regulators and biomarkers in NKTCL progression, and the expression level of MST1 and YAP may be closely related to the pathological development of NKTCL. Increasing MST1 or inhibiting YAP expression through pharmacological intervention may be promising for treatment of NKTCL. Hippo signaling pathway may be a novel therapeutic target for NKTCL. In the future, the development of drugs that activate the Hippo signaling pathway may be of great significance for treating NKTCL.

## CONFLICT OF INTEREST

The authors declare that they have no conflict of interest.
